# Methodology to Determine Melt Pool Anomalies in Powder Bed Fusion of Metals Using a Laser Beam by Means of Process Monitoring and Sensor Data Fusion

**DOI:** 10.3390/ma15031265

**Published:** 2022-02-08

**Authors:** Jana Harbig, David L. Wenzler, Siegfried Baehr, Michael K. Kick, Holger Merschroth, Andreas Wimmer, Matthias Weigold, Michael F. Zaeh

**Affiliations:** 1 Institute for Production Management, Technology and Machine Tools, Technical University of Darmstadt, 64289 Darmstadt, Germany; h.merschroth@ptw.tu-darmstadt.de (H.M.); m.weigold@ptw.tu-darmstadt.de (M.W.); 2 Institute for Machine Tools and Industrial Management, Technical University of Munich, 85748 Garching, Germany; siegfried.baehr@tum.de (S.B.); michael.kick@tum.de (M.K.K.); andreas.wimmer@iwb.tum.de (A.W.); michael.zaeh@iwb.mw.tum.de (M.F.Z.)

**Keywords:** additive manufacturing, multi-monitoring, PBF-LB/M, spatter

## Abstract

Additive manufacturing, in particular the powder bed fusion of metals using a laser beam, has a wide range of possible technical applications. Especially for safety-critical applications, a quality assurance of the components is indispensable. However, time-consuming and costly quality assurance measures, such as computer tomography, represent a barrier for further industrial spreading. For this reason, alternative methods for process anomaly detection using process monitoring systems have been developed. However, the defect detection quality of current methods is limited, as single monitoring systems only detect specific process anomalies. Therefore, a new methodology to evaluate the data of multiple monitoring systems is derived using sensor data fusion. Focus was placed on the causes and the appearance of defects in different monitoring systems (photodiodes, on- and off-axis high-speed cameras, and thermography). Based on this, indicators representing characteristics of the process were developed to reduce the data. Finally, deterministic models for the data fusion within a monitoring system and between the monitoring systems were developed. The result was a defect detection of up to 92% of the melt track defects. The methodology was thus able to determine process anomalies and to evaluate the suitability of a specific process monitoring system for the defect detection.

## 1. Introduction

Powder Bed Fusion of Metals using a Laser Beam (PBF-LB/M) is an additive manufacturing process. It is becoming increasingly important in industrial applications due to the high geometric freedom of the component design and the resource efficiency of the process. In recent years, it has been applied for components in highly stressed engineering applications [1]. These include, for example, turbine blades for aircraft engines or pistons for sport car combustion engines. These components must withstand high forces and temperatures during their use. They are therefore subject to strict standards and quality requirements. However, in the PBF-LB/M process, fluctuations appear that can affect the component quality.

Common defects in PBF-LB/M, which can result from process instabilities, are, for example, cavities (pores). These can be divided into different types according to their formation mechanism [2]. Most pores result from the inclusion of gas into the melt pool, and are therefore called gas pores. The gas originates from the surrounding atmosphere, such as the shielding gas [3]. It can also be released from gas inclusions within the powder [4]. The inclusions result from the gas atomization production of the powder or from spatter particles, which can appear in reused powder [5]. Larger gas pores result from instabilities in the keyhole. When the keyhole collapses, the surrounding atmosphere can be entrapped, resulting in the formation of a pore [6,7]. In addition to gas pores, a lack of fusion can also cause cavities. These elongated binding defects typically result from an insufficient energy input. The low energy input can cause the melt track to break off, creating a gap (balling). It was found that an increased number of spatters form when balling occurs during the process [8]. Therefore, the number of spatters and the geometry of the melt pool are potential criteria for the stability of the process. The influence of the porosity on the component properties depends on the shape, the size, and the location of the pores in the component [9,10]. Compared to elongated binding defects, spherical gas pores have a lower influence on the static mechanical properties [11]. This makes it necessary to employ quality assurance measures to meet the quality requirements for the process and for the components.

Since the PBF-LB/M process is mainly used for a small batch production, statistical quality assurance is difficult to guarantee [12]. Quality assurance methods based on in situ process monitoring are therefore increasingly subjects of research. Process monitoring systems can be used to detect process anomalies and defect-causing process conditions as early as possible in the process. Compared to current costly and time-consuming post-process measures for quality assurance, such as computed tomography, these systems can thus help to reduce production time and production rejects. For process monitoring in the PBF-LB/M process, the melt pool is of particular interest. Its geometry and dynamics are strong indicators for the stability and continuity of this process [13].

Process monitoring systems can be classified into on-axis and off-axis configurations. An on-axis system measures the process emissions following the beam path of the laser, while an off-axis system monitors the process from outside of the beam path of the laser. The on-axis systems typically employ pyrometers or high-speed cameras [14,15]. These systems are especially suitable for the characterization of the melt pool during the process, as the field of view moves with the melt pool. The off-axis systems often use thermographic cameras to monitor process by-products (e.g., spatters and fumes) [8,16,17,18,19]. Spatter tracking from frame to frame can increase the signal-to-noise ratio compared to the absolute number of spatters per frame [20]. Kolb et al. [21] used a combination of a high-resolution camera and a photodiode for an in situ surface evaluation of the PBF-LB/M process. Forien et al. [22] used a pyrometer to evaluate the keyhole stability for pore detection.

Each sensor, off-axis and on-axis, detects only specific process anomalies [23]. Due to this, the significance of a single sensor for a global defect detection is limited. The evaluation of the collected data is additionally dependent on the process knowledge of the user. Therefore, a comprehensive in situ quality assurance must monitor the process at different scales at the same time. The sensor data fusion allows combining the collected data to fulfill this need. Zhang et al. [24] used machine learning for the fusion of sensor data to detect flaws in PBF-LB/M single tracks. Petrich et al. [25] applied this method to multi-modal sensor data for the defect detection for PBF-LB/M processed components. However, the machine learning approach needs a high amount of experimental data for the training of the neural network.

In this work, a methodology for the sensor data fusion based on a small amount of experimental data is presented. With this methodology, it is possible to evaluate the quality and the suitability of a specific process monitoring system for the defect detection. The methodology was tested and validated by single melt track experiments in two PBF-LB/M systems with on-axis and off-axis process monitoring.

## 2. Materials and Methods

### 2.1. Research Approach

**Figure 1:**
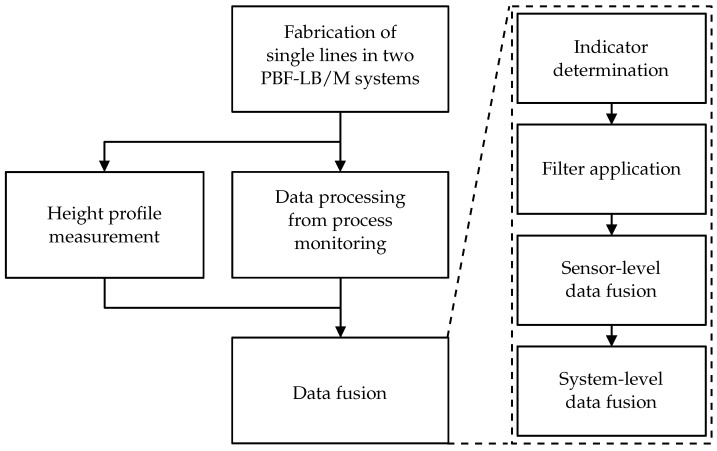
Research approach for the implementation of a data-fusion-based quality assurance for the PBF-LB/M process.

In this work, the influence of process anomalies on the quality of three PBF-LB/M melt tracks was investigated. The melt tracks were manufactured on two PBF-LB/M systems. Subsequently, the quality of the melt tracks was examined using a height profile from optical microscopy. Thereby, the regions with a severely reduced height in the melt track were assigned to balling defects. Process monitoring systems observed the manufacturing process. For this purpose, photodiodes, high-speed cameras, and a thermographic camera were used. The raw sensor data were mapped with the melt pool position within a single melt track and condensed to indicators. They are selected based on known process phenomena linked to defect-related process anomalies. To detect the anomalies, filters for the indicator signals were developed, calibrated, and evaluated. Afterwards, a two-step data fusion combined the data of the process monitoring systems. First, the indicators within each process monitoring system were combined on the sensor-level. Second, the indicators from the different process monitoring systems were linked by the subsequent data fusion on the system-level. The research approach is shown schematically in Figure 1.

### 2.2. Experimental Set-Up

The presented approach was examined in two experimental systems. Different process monitoring systems (on-axis and off-axis) and two materials (316L stainless steel and Scalmalloy) were used in the PBF-LB/M systems.

#### 2.2.1. EOS M290 and Monitoring Set-Up

**Figure 2:**
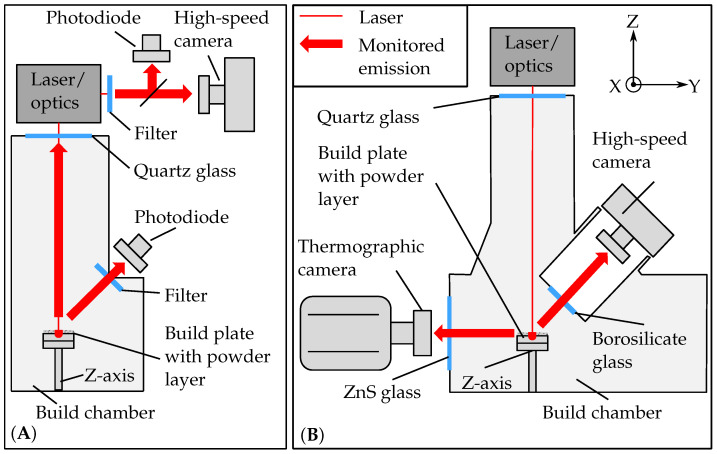
PBF-LB/M systems with process monitoring: (**A**) EOS M290 and (**B**) test bench.

The first PBF-LB/M system was an EOS M290 (Oxford, MA, USA) equipped with a 400 W fiber laser and a beam diameter of 100 μm (see Figure 2A). The material 316L stainless steel and a parameter set developed in previous studies were used for the fabrication of the melt tracks [26]. The applied monitoring system EOSTATE Melt Pool Monitoring (MPM) (Krailling, Germany) consisted of two photodiodes. One photodiode was positioned on-axis and detected the thermal radiation of the area where the laser was located. The second photodiode was positioned off-axis in the upper part of the process chamber and detected the entire layer. An optical band-pass filter in the range of 400–900 nm protected the photodiodes from backscattered laser radiation [27]. The second applied monitoring system was an on-axis high-speed camera (HSC1; plasmoEye, plasmo Industrietechnik, Wien, Austria). An optical band-pass filter limited the radiation to (900 ± 50) nm. The HSC1 recorded the thermal radiation with a frame rate of 13,800 Hz and a resolution of 160 × 140 pixels. The data from both process monitoring systems were assigned to the position in the melt track. For this purpose, the control position of the scanner mirrors was used. Due to the large amount of data, this assignment was performed ex situ.

#### 2.2.2. Test Bench and Monitoring Set-Up

The second PBF-LB/M system was a novel test bench for process monitoring (see Figure 2B). The energy source was a 1000 W fiber laser (YLR-1000-WC-Y14, IPG, Burbach, Germany) with a spot size of 80 μm. The optical instruments of the beam path were mounted on a breadboard (Thorlabs, München, Germany). The beam was expanded by a collimator (D50-F200, IPG, Burbach, Germany) and was then guided through the scanning system for area irradiation (intelliSCAN III30, Scanlab, München, Germany) and beam waist variation (varioSCAN de40i, Scanlab, München, Germany). A more detailed description of the test bench can be found in the work of [28]. The process monitoring system consisted of an off-axis thermographic camera (TC) and an off-axis high-speed camera (HSC2). The thermographic camera (X69000sc, FLIR Systems, Wilsonville, OR, USA) had a spectral range of 2 μm to 5 μm. It was equipped with a camera lens with 100 mm focal length. To increase the imaging scale, distance rings with a length of 68.5 mm were used. The TC was mounted in front of the build chamber to observe the process zone in the X-Z-plane. The observed window of the TC was resolved with 640 × 120 pixels at a frame rate of 3940 Hz. The HSC2 (Chronos 1.4, Kron Technologies, Hessen, state abbreviation, USA) faced the X-Y-plane of the process zone at an angle of approximately 60°. The process was monitored with a frame rate of 4532 Hz and a resolution of 1280 × 240 pixels. The data processing was performed ex situ. The start and end point of the laser were used to assign the camera data to the position in each melt track. The material Scalmalloy^®^ was used and processed with a scanning velocity of 900 mm/s, a laser power of 450 W, and a layer thickness of 80 μm.

### 2.3. Ex-Situ Melt Track Defect Detection

**Figure 3:**
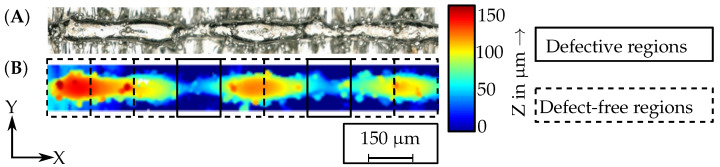
(**A**) Microscopy image of a single PBF-LB/M melt track and (**B**) exemplary detection of *reference defects* in a measured height profile of a single PBF-LB/M melt track.

An optical microscope (Infinite Focus, Alicona, Austria) was used to detect the balling defects in the manufactured melt tracks. These defects served as the *reference defects* for the defect detection in this work. An exemplary evaluated melt track is shown in Figure 3. The anomalies detected by the process monitoring systems are named *sensor defects* within this work. The height profiles of the melt tracks relative to the build plate were measured and processed using MATLAB. Sintered powder particles on the surface of the melt track led to noise in the signal. These data points were detected and excluded by an edge detection algorithm. To evaluate the defects quantitatively, deviations of the melt track height were detected. These deviations refer to the defect type balling in this method. A balling defect is regarded as the combination of a material accumulation and a following material-reduced area. The material accumulations are mainly a result of the surface tension of the melt [29]. The material reduced areas represent the actual defect as the continuous melt track breaks off. Within the melt tracks investigated in this work, such a defect pattern appears when the melt track height deviates more than 40 percent from the mean value of the melt track height. Regions with a melt track height more than 40% lower than the mean value were therefore defined as *reference defects*.

To quantitatively evaluate the defect detection capability of the monitoring systems, the melt tracks were divided into regions. In this work, the sizes of these regions depended on the data sampling rates of the individual process monitoring systems, as every region must contain several data points from each process monitoring system. The width of the regions in X-direction for the EOS M290 was 125 μm and 300 μm for the test bench in this work. The regions with a defect were called *defective regions* and the regions without a defect were called *defect-free regions*.

## 3. Data Fusion

The area surrounded by dashed lines in Figure 1 shows the individual steps of the data fusion. The process monitoring data is first reduced to signals of individual indicators. Using filters, defect-related anomalies are found in the reduced indicator signal. The data is then fused for each individual sensor (sensor-level data fusion). Afterwards, the data of the different process monitoring systems are fused (system-level data fusion). The following section describes the methodology of the individual steps in detail.

### 3.1. Indicator Determination

The indicators are characteristics within the signal of the process monitoring systems, such as the width of the melt pool in a camera signal. Indicators thus reduce the signal to a minimum amount of data without a loss of information. The raw data of the process monitoring system is analyzed qualitatively to determine these indicators. Based on the understanding of defect-related process phenomena (e.g., the collapse of the melt pool), a pre-selection of indicators is possible. Additionally, the data of the process monitoring systems can be compared qualitatively to the *reference defects*. This makes it possible to find further anomalies in the data from the process monitoring systems, which then serve as indicators.

### 3.2. Filter

**Figure 4:**
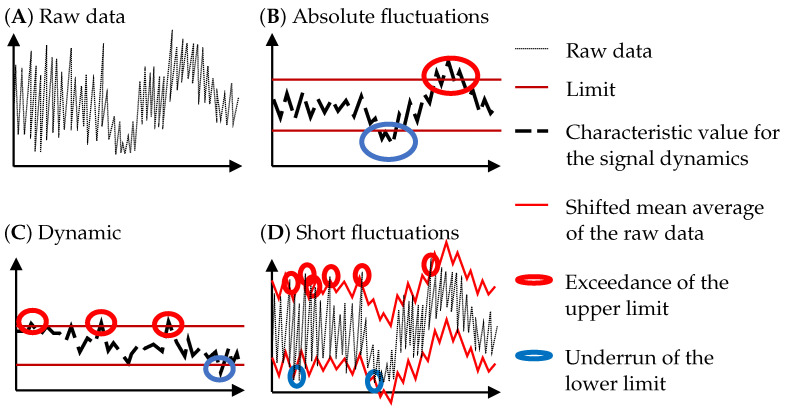
Filter algorithms for the detection of anomalies in the PBF-LB/M process.

In the previous step, the indicators are determined to reduce the raw process monitoring data. In order to detect the anomalies, it is necessary to identify deviations in the indicator signal. For this purpose, three different filter algorithms are developed within this work (see Figure 4). The first algorithm detects *absolute fluctuations* around the mean value of the whole signal of the melt track (see Figure 4B). The mean value of the data series is therefore calculated. A calibration factor shifts the mean value up and down. The shifted signal defines the upper and the lower threshold for the allowed signal deviation. A region is defined as *sensor defective*, if the signal value of this region is outside of these thresholds. Thereby, the *sensor defects* are classified according to the threshold (upper or lower) that is passed. The second filter algorithm detects anomalies in the signal dynamics (see Figure 4C). The difference in the signal value of two sequencing data points specifies the dynamics of the signal. Afterwards, the calculated dynamics are analyzed for absolute fluctuations in a way analogous to the filter algorithm for *absolute fluctuations*. The last filter algorithm detects *short fluctuations* (see Figure 4D). A moving average of the signal is calculated and then shifted up and down by a calibration factor. These two curves then form the thresholds for the signal.

### 3.3. Filter Calibration

To design and evaluate the defect detection of the process monitoring systems, the defects detected by the filters (*sensor defects*) must be compared with the *reference defects*. If the filter shows a deviation at least once over the length of a defective region, the monitoring system detects the *reference defect* correctly. A defect-free region is correctly detected if the signal permanently stays between the thresholds of the filter. Table 1 gives an overview of this defect evaluation.

Here, *TP* is the number of the correctly identified defective regions, *TN* is the number of the correctly identified defect-free regions, *FP* is the number of the incorrectly identified defective regions, and *FN* is the number of the incorrectly identified defect-free regions. With this defect evaluation, the filters are calibrated for each indicator signal separately. If the values for the *sensitivity* and the *specificity* are on the same level within ±10%, the calibration is considered to be successful in this work. The *sensitivity* and the *specificity* are calculated by the following equations:

(1) sensitivity=TP/(TP+FN),

(2) specificity=TN/(TN+FP).

In the remainder of this paper, these values are given with the notation (*sensitivity*|*specificity*). Based on this, the data fusion is performed for each type of defect to further increase the accuracy of the defect prediction. In particular, the sensor data fusion accounts for the suitability of each process monitoring system for the detection of specific causes of defects.

### 3.4. Sensor-Level Data Fusion

Each filter–indicator combination is particularly suitable for the detection of a specific type of defect cause. A fusion of these data enables an extension, and thus an improvement, of the detection. The *accuracy* value of each combination can be used as an evaluation parameter for the system-level data fusion. The *accuracy* is calculated by:

(3) accuracy=(TP+TN)/(TP+TN+FP+FN).

**Figure 5:**
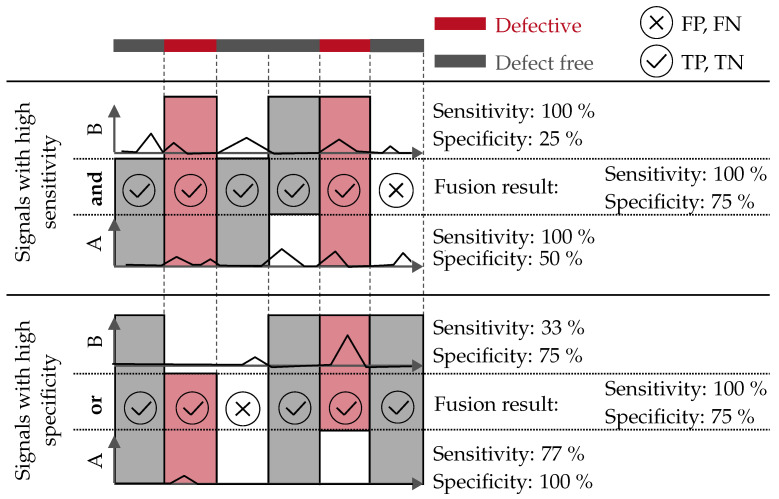
Principle of the sensor-level data fusion based on the *sensitivity* and *specificity* of two exemplary signals A and B.

For the further evaluation, only filter–indicator combinations with an *accuracy* above 65% are considered to exclude purely statistical detections within the scope of this work. The remaining combinations are then sorted according to the *sensitivity* and the *specificity*. The combinations with a high *sensitivity* are linked with the logical operator “and” to improve the *specificity*. This means, that the fused signal indicates a *sensor defect*, if both signals indicate a *sensor defect*. Otherwise, it is marked as *sensor defect-free*. The signals with a high *specificity* are linked with the logical operator “or” to increase the *sensitivity*. Here, the fused signal indicates a *sensor defect*, if one or both signals indicate a *sensor defect*. If both signals are *sensor defect-free*, the result is also *sensor defect-free*. The principle of this data fusion is shown in Figure 5.

### 3.5. System-Level Data Fusion

The sensor-level data fusion results in a sensitivity and specificity value for each individual process monitoring system. These values provide information about the prediction quality of the systems. The different process monitoring systems can only detect specific defects and defect causes due to their specification. The detection quality can be increased by the same method as for the sensor-level data fusion. In the case of different detection qualities of the individual monitoring systems, the fusion of a system with a high prediction quality with a system with a significantly lower prediction quality leads to no improvement. High deviations of the prediction qualities lead to a deterioration of the overall system.

## 4. Results and Discussion

The data fusion methodology presented in Section 3 was applied to process monitoring data of single melt track experiments. The experiments were conducted in two PBF-LB/M systems. In this section, the defect detection capability of the systems is presented. First, the indicators and the filter combinations for the systems are described. Second, the results of the sensor-level data fusion and the data fusion on the system-level are presented.

### 4.1. Indicators and Filters

#### 4.1.1. EOS M290

**Figure 6:**
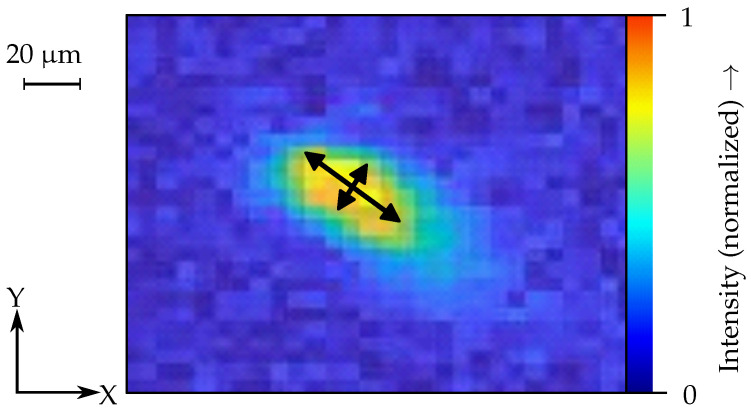
Indicators for the melt pool geometry for the monitoring system HSC1.

The MPM provides one intensity data set each for the on-axis photodiode and for the off-axis photodiode. These intensity data sets were used directly as indicators, as they cannot be reduced any further. Additionally, the HSC1 provides the spatially resolved 2D intensity distribution of the melt pool. Important indicators for the process stability are the length and the width of the melt pool (see Figure 6). This is due to the interruption of the melt pool when balling occurs. During the interruption and rebuild of the melt pool, a reduction of its length and width can be expected. In addition to the geometry, the mean and the maximum intensity of the melt pool were measured. The mean intensity was used as an indicator for relative changes in the overall temperature of the melt pool. The variations of the maximum intensity were used as an indicator for changes of the temperature gradient. However, only qualitative conclusions from the intensity to the temperature were possible since the emission coefficient of the molten material was unknown. The two indicator systems, the melt pool geometry and the melt pool intensity, are related to different process phenomena. Hence, they are considered as two individual systems in the following.

Due to the dynamics of the molten material, the rule for the detection of a *sensor defect* was adapted during the analysis of the data. The measurement data showed that the position of a process anomaly and the position of the resulting defect were not always the same. It is supposed that this is related to the contraction of the molten material during solidification. If a filter detects an anomaly in the surroundings of a *defective region*, it counts as a correctly detected *sensor defect*.

Falling below the lower limit in the filter *absolute fluctuations* led to the highest *sensitivity* and *specificity* values for the indicators melt pool width (61|77), melt pool length (72|73), maximum intensity of the HSC1 (68|76), mean intensity of the HSC1 (64|86), and intensity of the on-axis photodiode (71|74). The filter *short fluctuations* was not suitable for the defect detection. The accuracy values were below 60% for all indicators. The filter *dynamic* showed increased values when an accumulation of material occurred. The increased values were found for the indicators intensity of the on-axis photodiode, melt pool length, and melt pool width. This was attributed to the interruption and reconstruction of the melt pool when balling occurs. Thus, the process monitoring signal shows increased signal dynamics in this region. However, the material accumulations were not considered as a defect in this work.

#### 4.1.2. Test Bench

**Figure 7:**
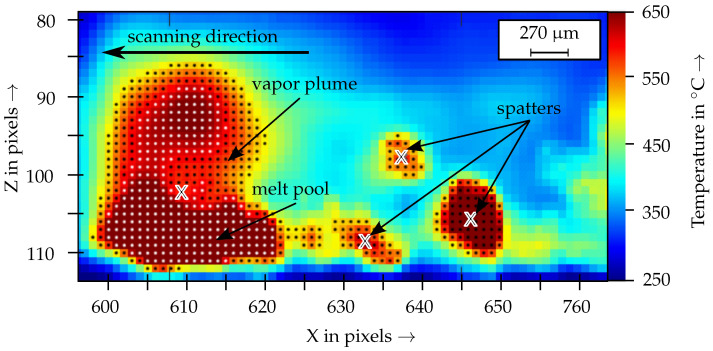
Indicators for the melt pool geometry (white dots) and for the number of spatters (black dots) in the monitoring system TC; the centroids of the melt pool and of the spatters are marked with an “X”.

The data of the TC were reduced to two different indicator systems: the melt pool geometry and the number of spatters. As described above, using the number of spatters as an indicator for the process stability can be beneficial, as a high number of spatters implies strong melt pool dynamics, and thus an unstable process. Similarly, the melt pool geometry can indicate defect phenomena such as balling. The height of the centroid of the melt pool and the total pixel area were selected as indicators for the melt pool geometry (see Figure 7). The threshold method was used to detect the spatters and the melt pool in each frame. Here, different threshold values were used for the two indicator systems to clearly separate them. It was assumed, that the melt pool always describes the largest contiguous area of pixels in each frame. Subsequently, the coordinates of the center of gravity and the total area of the detected pixels were determined. To improve the signal-to-noise ratio of the spatter detection, the spatters were only included in the evaluation if they were tracked over four consecutive frames. For this purpose, a spatter tracking algorithm similar as in the work of [20] was used and adapted for the analysis of the TC data. In addition to the number of spatters, the pixel area of the largest spatter per frame was also included as an indicator. For the HSC2, only the total number of spatters was chosen as an indicator, as the melt pool was not resolved in detail.

Due to the low sampling rate of the process monitoring systems in the test bench compared to the systems in the EOS M290, only defects lager than 50 μm were detected. The indicators of the melt pool geometry showed the highest *sensitivity* and *specificity* values with exceedance of the upper limit in the filter *absolute fluctuations* for the melt pool height (77|74) and for the melt pool area (77|88). With the filter *short fluctuations* values of (77|76) for the melt pool height and (77|72) for the melt pool area were achieved. In the case of the number of spatters, falling below the lower limit in the filter *dynamic* (55|74) and in the filter *short fluctuations* (77|62) showed the best results. For the indicator area of the largest spatter, exceedance of the upper limit in the filters *dynamic* (77|60) and *absolute fluctuations* (77|64) and falling below the lower limit in the filter *short fluctuations* (77|80) were most suitable for anomaly detection. The defect detection capability of the HSC2 showed significantly lower accuracy for the detection of *reference defects* for all indicator–filter combinations. Here, falling below the lower limit in the filters *dynamic* (77|49) and *short fluctuations* (55|47) were the most suitable.

### 4.2. Sensor-Level Data Fusion

#### 4.2.1. EOS M290

The sensor-level data fusion improved the prediction accuracy for a specific anomaly, especially for the HSC1. The low melt pool width and the low melt pool length in the filter *absolute fluctuations* were fused with the logical operator “or” (77|76). The fusion of the low average and the low maximum intensity in the filter *absolute fluctuations* resulted in values of (77|76). These results showed that the reduction in the melt track volume is also accompanied by a reduction of the melt pool area. The reduction in the intensity indicated that the temperature in the melt pool was also reduced. However, this can only be stated as a hypothesis, since the emission coefficient of the molten material changes nonlinearly with the temperature [30]. The reduction of the melt pool dimensions and the reduction of the melt pool intensity superimposed each other in the intensity signal of the MPM. Both reduced the recorded intensity data of the photodiodes.

#### 4.2.2. Test Bench

As with the EOS M290 process monitoring systems, the data fusion led to a significant increase in the defect prediction accuracy of the test bench. For the indicator melt pool area, a data fusion with the logical operator “or” increased the statistical values to (100|72). The data fusion within the indicator melt pool height led to statistical values of (78|82). For the indicator total amount of spatters, falling below the lower limit of the filters *dynamic* and *short fluctuations* were fused with the logical operator “or” (89|54). Higher values were achieved for the number of large spatters. The exceedance of the upper limit of the filters *short fluctuations* and *dynamic* were fused with the logical operator “or”. With this sensor-level data fusion, values of (89|71) were achieved. These results indicated, that a separation of a large amount of molten material (large spatter) from the melt pool results in a *reference defect* in a single melt track. Simultaneously, the total number of spatters decreases. Before the large spatter separates, an enlargement of the melt pool in the Z-direction can be measured. In this work, the mean area of the melt pool was 150 pixels and the mean centroid Z-position was 10.6 pixels. A defect was indicated for melt pools with an area greater than 200 pixels and a centroid Z-position higher than 12 pixels. This corresponds to an enlargement of the melt pool area of 33.3% and an increase of the centroid Z-position of 13.2%. This behavior could be attributed to the collapse of the keyhole of the melt pool and hence to the formation of a sub-surface pore in the resulting component [31].

The data of the HSC2 showed lower values during the evaluation of the filters. The sensor-level data fusion improved these values, but they were still below the values of the other process monitoring system of the test bench. The fusion of falling below the lower limit in the filters *dynamic* and *short fluctuations* for the total number of spatters with the logical operator “and” resulted in values of (56|70).

### 4.3. System-Level Data Fusion

#### 4.3.1. EOS M290

The system-level data fusion of the different process monitoring systems improved the overall *sensitivity*. However, the *specificity* decreased slightly. For the detection of the defects for the single melt tracks, values of (92|67) were achieved. This accounted for an increase in the *sensitivity* of 15% compared to the sensor-level data fusion. Therefore, the HSC1 melt pool geometry, the HSC1 melt pool intensity, and the MPM signal of the on-axis photodiode out of the sensor-level fusion were combined with the logical operator “or”.

#### 4.3.2. Test Bench

In this study, the HSC2 and the TC showed very different prediction qualities of (56|70) and (89|86), respectively. A fusion would have reduced the prediction accuracy of the merged system compared to the data of the TC. Due to this, the data of the two systems were not fused. The values for the *sensitivity* and *specificity* of the TC were achieved with the fusion of the TC melt pool geometry and the TC spatters by the logical operator “and”.

## 5. Conclusions and Outlook

In this work, a methodology for improved defect detection in the PBF-LB/M process by sensor data fusion was presented. The methodology was applied to the balling defect type as an example. First, indicators were defined based on process knowledge about defect causes and a qualitative analysis of the data. Using the threshold filters *absolute fluctuations*, *dynamic*, and *short fluctuations*, process anomalies were identified. Via a quantitative evaluation by the statistical parameters *sensitivity* and *specificity*, the filters were calibrated. Finally, a two-stage data fusion was applied. On the sensor-level, the characteristics of the three filters of each process monitoring system were fused with logical operators. Afterwards, the different process monitoring systems were fused according to the sensor-level data fusion. From the results of this work, the following conclusions can be drawn:

- The presented methodology enabled the defect detection in single melt tracks manufactured on two different PBF-LB/M systems. They differed in terms of the machine, the process monitoring systems, and the material. For the EOS M290 with 316L powder, values of (*sensitivity*: 92|*specificity*: 67) were achieved. The test bench with Scalmalloy^®^ powder showed values of (*sensitivity*: 89|*specificity*: 86).
- In both PBF-LB/M systems, the data fusion enabled a significant increase of up to 20% in the *sensitivity* of the defect detection. It was shown that each process monitoring system detects different defect related process phenomena. The fusion of the data enabled a more comprehensive evaluation of the causes of the defects.
- A reduction in the dimensions of the melt pool and in the intensity of the melt pool were suitable indicators for the defect detection with on-axis process monitoring systems. These systems can detect a melt pool collapse through the correlated short-term reduction in the melt pool size and the cooling of the molten material.
- Off-axis systems showed the melt pool in the X-Z and in the X-Y plane and allowed for a larger image section. Viewing in the X-Z plane allowed the extension of the melt pool in the Z-direction to be observed. Melt pools with an area greater than 200 pixels (mean area = 159 pixels) and a Z-position of the centroid higher than 12 pixels (mean Z-position = 10.6 pixels) indicated a defect. Off-axis systems with a large field of view enabled the detection of spatters, but had a reduced acquisition rate. Nevertheless, these systems showed dynamics in the melt pool as it collapsed. This was detected by the separation of large spatters from the melt pool.

In future work, the methodology should also be applied to 2D melting surfaces and 3D solids. This will extend the investigations to further defect types, such as porosity. Additionally, the methodology can be extended to a classification of the defect type and to a quantification of the defect size. For this purpose, the magnitude of a *sensor defect* can be evaluated by the value of the detected process anomaly. In parallel to this consideration, an optimization of the methodology should be carried out, which examines and optimizes the influence of the decision rules applied within this study. This work showed that the different process monitoring systems of the test bench and the EOS M290 detected different process anomalies. The fusion of these systems would enable more comprehensive detection of defect-related process anomalies and could further improve the defect detection and evaluation.

## Figures and Tables

**Table 1:** Fourfold table of the defect evaluation.

	Reference Defect	No Reference Defect
Defective region	*TP*	*FP*
Defect-free region	*FN*	*TN*

## Data Availability

The data presented in this study are available on request from the corresponding authors.
